# Plasma levels of angiopoietin-1 and -2 predict cerebral malaria outcome in Central India

**DOI:** 10.1186/1475-2875-10-383

**Published:** 2011-12-23

**Authors:** Vidhan Jain, Naomi W Lucchi, Nana O Wilson, Anna J Blackstock, Avinash C Nagpal, Pradeep K Joel, Mrigendra P Singh, Venkatachalam Udhayakumar, Jonathan K Stiles, Neeru Singh

**Affiliations:** 1 Regional Medical Research Center for Tribals (ICMR), Nagpur Road, Garha, 482003 Jabalpur, Madhya Pradesh, India; 2 Atlanta Research and Education foundation Decatur, GA, USA; 3 Malaria Branch, Division of Parasitic Diseases and Malaria, Center for Global Health, Centers for Disease Control and Prevention (CDC), Atlanta, GA, USA; 4 Department of Microbiology, Biochemistry and Immunology, Morehouse School of Medicine, Atlanta, GA, USA; 5 Nethaji Subhash Chandra Bose Medical College Hospital, Jabalpur, Madhya Pradesh, India; 6 National Institute of Malaria Research Field Unit (ICMR), Jabalpur, Madhya Pradesh, India

**Keywords:** Angiopoietins, Cerebral malaria, Pathogenesis, Biomarkers, Receiver operating characteristic analysis

## Abstract

**Background:**

The mechanisms underlying the pathogenesis of cerebral malaria (CM) syndrome are not well understood. Previous studies have shown a strong association of inflammatory chemokines, apoptotic markers and angiogenic molecules with CM associated mortality. Recognizing the importance of angiopoietins (ANG) in the pathogenesis of CM, a retrospective investigation was carried out in a hospital cohort of malaria patients with *Plasmodium *infection in central India to determine if these factors could be suitable markers of CM associated severity.

**Methods:**

Patients enrolled in the study were clinically characterized as healthy controls (HC), mild malaria (MM), CM survivors (CMS) and CM non-survivors (CMNS) based on their malaria status and hospital treatment outcome. Plasma ANG-1 and ANG-2 levels were assessed using sandwich ELISA. Receiver operating characteristic (ROC) curve analysis was used to calculate area under the curve (AUC) for each biomarker in order to assess predictive accuracy of individual biomarkers.

**Results:**

The plasma levels of ANG-1 were lower in CMS and CMNS compared to control groups (mild malaria and healthy controls) at the time of hospital admission. On the contrary, ANG-2 levels positively correlated with malaria severity and were significantly higher in CMNS. The ratio of ANG-2/ANG-1 was highest in CMNS compared to other groups. Receiver operating characteristic curves revealed that compared to ANG-1 (AUC = 0.35), ANG-2 (AUC = 0.95) and ratio of ANG-2/ANG-1 (AUC = 0.90) were better markers to discriminate CMNS from MM cases. However, they were less specific in predicting fatal outcome amongst CM cases at the time of hospital admission.

**Conclusion:**

These results suggest that at the time of admission plasma levels of ANG-2 and ratio of ANG-2/ANG-1 are clinically informative biomarkers to predict fatal CM from MM cases while they have limited usefulness in discriminating fatal CM outcomes in a pool of CM cases in endemic settings of Central India.

## Background

Cerebral malaria (CM) is a severe form of central nervous system (CNS) pathology associated with *Plasmodium falciparum *infection. It is characterized by unarousable coma that often begins with seizures among children but coma in adults is less frequently associated with seizures [1]. Despite treatment, mortality due to CM can be as high as 30%, while neurological sequelae that are uncommon in adults occurred among 10% of children recovering from CM [1-3]. Further CM is also associated with cognitive deficit [4,5]. Early diagnosis and prompt treatment can minimize or avert mortality and morbidity associated with CM. The mechanisms underlying the pathogenesis of this multi-factorial syndrome are unclear.

Sequestration of parasitized red blood cells (PRBCs), mainly late trophozoite and schizonts, within the microvasculature (capillaries and post capillary venules) are thought to play an important role in the pathogenesis of CM [6]. It has also been proposed that downstream events following sequestration, such as dysregulation of the immune system (primarily by over-production of inflammatory factors such as TNF-α, lymphotoxins, IFN-γ and its inducible protein CXCL10/IP-10) may play an important role in the pathogenesis [7-10]. Parasite-induced soluble factors may contribute directly to a breach in the blood brain barrier (BBB) and neuronal pathology, possibly via apoptotic pathways [11].

Platelets (regulators of haemostasis) have also been considered as effectors of CM pathogenesis. Binding of platelets and platelet microparticles (PMP) (facilitated on one hand by sticky von-Willebrand factor [vWF] exposed on activated endothelium and on another with PRBCs through receptors CD-31 and CD-36) may promote cytotoxicity to the TNF and LT-α activated brain endothelial cells (EC) [12,13]. As evidenced from these studies, the acute and advanced phases of CM are thought to be associated with endothelial sequestration, inflammation and hemostatic disorder leading to microcirculatory dysfunction [14].

Previous studies carried out among Indian CM patients have shown that severe malaria patients who died of CM had significantly lower plasma levels of angiogenic factors such as vascular endothelial growth factor (VEGF) and platelet derived growth factor (PDGFbb) [10,15]. Other angiogenic factors such as angiopoietins (ANG) have recently been investigated among African children and South East Asian adults to test their utility as potential functional biomarkers for severe malaria [15].

ANG-1 is a vascular quiescence molecule whereas ANG-2 is an antagonist of ANG-1 by binding to the common receptor Tie-2 [16]. ANG-2 primes the endothelium to respond to exogenous stimuli and facilitates the activities of inflammatory factors (TNF and IL-1) and angiogenic factors like VEGF and PDGFbb [17]. Recent studies have reported different levels of specificity and sensitivity in using ANG-1, ANG-2 and ANG-1/ANG-2 ratio for discriminating CM patients from other malaria patients [18-21].

VEGF is an important factor that induces angiogenesis and vasculogenesis. Interactions of angiopoietins with VEGF promote angiogenesis, whereas in the absence of VEGF, ANG-2 causes regression of blood vessels [16]. To better understand the role of ANG-1 and ANG-2 in CM outcomes in a different malaria endemic setting, a retrospective investigation was carried out in Central India. In addition, predictive values of ANG-1, ANG-2 and their ratios were also determined in order to test the utility of these factors in discriminating fatal CM from non-fatal CM and mild malaria patients.

## Methods

### Study background and site

Samples used in this study were collected as part of an NIH-funded research project (R21TW006804-01) from 2004 to 2007 at NSCB Medical College Hospital, Jabalpur and Civil Hospital Maihar, district Satna, both in the state of Madhya Pradesh, Central India. This study was approved by the ethical research committee of National Institute of Malaria Research New Delhi, Regional Medical Research Center for Tribals, Jabalpur, Center for Disease Control and Prevention, CDC, Atlanta, GA and Morehouse School of Medicine, Atlanta, GA, USA.

### Subjects and enrolment criteria

Children (< = 14 years) and adults with only *P. falciparum *positive asexual stage parasitaemia on blood smear were enrolled after obtaining written informed consent from their parents or close relatives. Consent was also obtained for long-term storage and later use of samples. Patients satisfying the enrolment criteria were enrolled as healthy control (HC), mild malaria (MM) and cerebral malaria (CM) following the definition given below. Malaria associated complications were defined using WHO criteria [1,22].

#### Healthy control

Relatives of patients in the hospital and members of the community who did not have malaria or other febrile illness 60 days before enrollment, and no past history of mental/metabolic illness, tuberculosis, meningitis, or accidental head injury were included.

#### Mild malaria

Patients who had fever with *P. falciparum *parasitaemia of < 25,000 parasites/μl of blood (detected microscopically from blood smears) and no evidence of severe malaria and no past history of mental/metabolic illness, tuberculosis, meningitis, or accidental head injury were included.

#### Cerebral malaria

To be considered a case of CM, a patient had to fulfill the WHO's definition of severe CM [1], have a Glasgow coma score of ≤ 10 [23] have a *P. falciparum *parasitaemia, and have no other clinically evident cause of impaired consciousness, no past history of mental/metabolic illness, tuberculosis, meningitis, or accidental head injury were included.

### Blood samples

Venous blood samples were obtained prior to treatment with intravenous quinine or α-β arteether. On the basis of treatment outcome, CM patients were further divided into two subgroups; CM survivors (CMS) and CM non-survivors (CMNS). Detailed description of patients has been described elsewhere [22].

### Detection of ANG-1 and ANG-2 levels

Plasma ANG-1 and ANG-2 levels were assessed by sandwich ELISA (R & D Systems, Minneapolis, MN) following manufacturer's guidelines. Briefly, NUNC maxisorb, ELISA plates were incubated overnight with 100 μl of diluted capture antibody for ANG-1/ANG-2 in PBS (pH = 7.2). This was followed by three washes with buffer (PBS pH 7.2 and 0.05% Tween 20). Blocking was done by 1% bovine serum albumin in PBS for 1 h. Recombinant proteins were serially diluted as recommended, to obtain a seven point standard curve. All the samples were diluted 1:4 in reagent diluent. Samples that showed higher OD than the highest standard were diluted further. Samples and standards were incubated for 1 h at room temperature (on shaker at 500 rpm), followed by washing and addition of detection antibodies, which were incubated for 1 h. After four washes, streptavidin-HRP was added and incubated for 20 min. The substrate tetra methyl benzidine (TMB)/H2O2 (Bangalore, Genie, India) was added and plates were incubated at room temperature in the dark for 20 min. Fifty microlitres of stop solution (2N H_2_SO_4_) were then added to each well and optical density (OD) was assessed at 450 nm.

### Sensitivity of immunoassay

Upper and lower limits of detection for each biomarker were: ANG-1, 10000 - 156.25 pg/mL and for ANG-2, 3500 - 54.69 pg/mL (according to manufacturer). Detection in our samples (diluted 1:4) ranged from 7527.6-71.9 pg/mL for ANG-1 and 3500-11.6 pg/mL for ANG-2.

### Statistical analysis

Statistical analyses were performed using STATA version 8.2 software (StataCorp, College Station, TX). Receiver operating characteristic (ROC) curve and area under the ROC curves (AUC) were generated. Continuous variables were analysed using the Kruskal-Wallis test. Angiopoietin levels and survival outcomes were analysed using the Wilcoxon rank-sum test. Categorical variables were analysed using Chi-square test. *P *value < 0.05 was considered significant. Cut-off values were derived mathematically from the ROC curves, using the ROC curve with the lowest value for the formula: (1-sensitivity)^2 ^+ (1-specificity)^2^. Logistic regression analysis was done in order to strictly define association of categorical severity indicators with mortality controlling for age and parasitaemia.

## Results

### Clinical characteristics of study subjects

The details of patients' characteristics and clinical parameters are given in Table 1. There were no significant differences between the patients' age, parasitaemia and gender (*P *> 0.05). The study population was comprised of both adults and children, wherein the Glasgow coma score for CMNS was significantly lower (*P *< 0.015) than CMS. Respiratory distress (*P *< 0.045) and seizure (*P *= 0.061) were found to be associated with CMNS than CMS. However, in multivariate logistic regression analysis (controlling for age and parasitemia) respiratory distress and seizure were not associated with mortality outcome in CM (*P *= 0.062 and 0.088 respectively). As expected, HC had significantly higher haemoglobin levels than MM (*P *< 0.002), CMS (< 0.0001) and CMNS (*P *< 0.0001). Plasma levels of angiopoietins were not affected by age, gender and parasitaemia in this study (*P *> 0.05). Among CM patients, respiratory complication, seizures and severe anaemia were not associated with a rise in plasma angiopoietins (*P *> 0.05).

**Table 1:** Clinical/parasitological characteristics and angiopoietins level of enrolled patients (HC = healthy controls, MM = mild malaria, CMS = cerebral malaria survivors and CMNS = cerebral malaria non survivors)

Variable	HC	MM	CMS	CMNS
Number	34	57	63	29
Age (IQR)	25 (14-32)	19 (12-36)	25 (12-40)	25 (13.5-37.5)
Children, n (%)	9 (26.4)	20 (35.1)	21 (33.3)	8 (27.6)
Gender (M/F)	23/11	29/28	44/19	18/11
Coma Score (IQR)	14	14	8 (6-9)	6 (4-8)
Haemoglobin (g/dl) (IQR)	11.6 (10-12.7)	9 (7.9-11.3)	8.1 (6-10)	6.8 (5.4-9.5)
Seizure (%)	0	0	20 (31.74)	15 (51.72)
Hypoglycaemia, n (%)	0	0	4 (6.34)	1 (3.44)
Renal failure, n (%)	0	0	15 (23.80)	8 (27.58)
Jaundice, n (%)	0	0	13 (20.63)	6 (20.68)
Hepatic Encephalopathy, n (%)	0	0	4 (6.34)	2 (6.89)
Respiratory failure, n (%)	0	0	10 (15.87)	10 (34.48)
Haemolysis, n (%)	0	0	7 (11.11)	1 (3.44)
Abnormal bleeding, n (%)	0	0	1 (1.58)	3 (10.34)
Hypotension, n (%)	0	0	11 (17.46)	7 (24.13)
Mild anaemia, n (%)	8 (23.5)	20 (35)	20 (31.74)	6 (20.68)
Moderate anaemia, n (%)	0	9 (15.7)	21 (33.33)	9 (31.03)
Severe anaemia, n (%)	0	2 (3.5)	7 (11.11)	6 (20.68)
Neurological sequelae, n (%) *Multiorgan dysfunction	0 0	0 0	5 (7.93) 10 (15.87)	0 8 (27.58)
ANG-1 (IQR)	12413.7 (6728.4-17590.4)	4644.4 (2508-8822.5)	2849.76 (1678.78-5211.32)	2359.61 (1582.31-5062.92)
ANG-2 (IQR)	335.9 (163.9-599.2)	968.7 (480.3-1948.9)	3580.73 (1193.59-7104.86)	7779.06 (5701.50-12197.17)
ANG-2/ANG-1 (IQR)	0.02 (0.01-0.05)	0.16 (0.08-0.47)	1.42 (0.41-2.91)	2.69 (1.06-7.04)
Parasites/300 WBCs (IQR)	0	44 (21-128)	28 (7-237)	42 (8-296)

Continuous variables were presented as median inter quartile range (IQR) and categorical variables were presented as numbers (%), except gender. Groups were compared using Kruskal-Wallis test (continuous variable) and Chi-square test (categorical variables)

*Multiorgan dysfunction is defined by combinations of 2 or more complications (respiratory distress, renal failure, jaundice, hypotension, gastrointestinal bleeding)

### ANG-1, ANG-2 and their ratios versus disease severity

**Figure 1:**
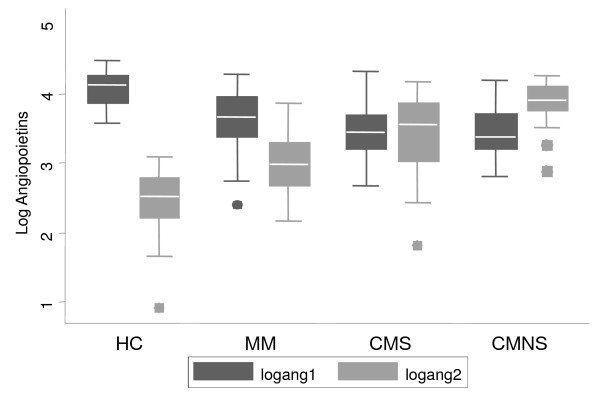
**Angiopoietins (ANG) were assessed using ELISA**. Plasma levels of ANG among studied groups (HC = healthy controls, MM = mild malaria, CMS = cerebral malaria survivors and CMNS = cerebral malaria non-survivors) are presented with box plots (25-75 percentile distribution of data) with bars and medians. Data shown as points are outliers.

Plasma levels of ANG-1 and ANG-2 among the 183 subjects were assessed. Plasma levels of ANG-1 declined with disease severity (Figure 1). Compared to HC, median plasma levels of ANG-1 significantly declined (2.7 fold) among MM subjects (*P *< 0.0001), 4.4 fold in CMS (*P *< 0.0001), and 5.3 fold in CMNS (*P *< 0.0001). As compared to MM, there was a 1.6-fold and 2-fold decline in ANG-1 levels in CMS (*P *< 0.0035) and CMNS groups (*P *< 0.025), respectively. However, ANG-1 levels were not significantly different between CMNS and CMS (*P *= 0.8). In contrast, the median plasma levels of ANG-2 progressively increased with disease severity (Figure 1). Compared to HC group, a 2.9-fold increase was observed in MM group (*P *< 0.0001), 11.3 fold in CMS (P < 0.0001) and 23.2 fold in CMNS (*P *< 0.0001). In comparison to MM, CMS and CMNS showed 3.9- (*P *< 0.0001) and 8-fold (*P *< 0.0001) increase, respectively. The median ANG-2 levels were significantly higher (2 fold) among CMNS than CMS (*P *< 0.0001).

The median ratios of ANG-2/ANG-1 progressively increased with disease severity (Table 1). Compared to HC group, an increase of 8 fold was observed in MM group (*P *< 0.0001), 71-fold increase in CMS (*P *< 0.0001) and 134.5-fold increase in CMNS (*P *< 0.0001) group. In comparison with MM, CMS and CMNS showed 8.8-fold (*P *< 0.0001) and 16.8-fold (*P *< 0.0001) increases, respectively. The median ratio was significantly higher (1.9 fold) among CMNS than CMS (*P *< 0.001).

### Receiver operating characteristics (ROC) curves

**Figure 2:**
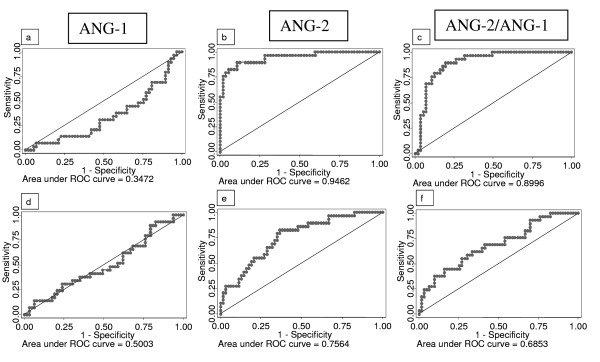
**Assessment of angiopoietins (ANG) utility in discriminating between fatal malaria with other groups using ROC analysis**. Receiver operating characteristic curves (bold lines) were generated for each factor to compare CMNS with MM patients (a, b, c) or CMNS with CMS (d, e, f). The null hypothesis (thin line) is that the area under the curve (AUC) equals 0.5. AUC for each curve is given just below the graphs.

Biomarkers that predict development of severe malaria outcome can be clinically utilized for prognosis, diagnosis and disease management. An additional analysis of data using ROC curves was undertaken to determine the cutoff value that discriminates between two groups with reasonable sensitivity and specificity. ROC curves were plotted to discriminate between CMNS versus MM and CMNS versus CMS patients. The area under curve (AUC) of ANG-1 for CMNS versus MM was 0.35 and for CMNS versus CMS 0.50 (Figure 2a, d).

The AUC of ANG-2 was shown to be 0.95 for CMNS versus MM and 0.76 for CMNS versus CMS (Figure 2b, e). A cut-off value of 3246.8 pg/ml was 89.7% sensitive and 89.5% specific in differentiating CMNS from MM patients while a cut-off of 4924.52 pg/ml was 82.8% sensitive and 63.5% specific in differentiating CMNS from CMS. The ANG-2/ANG-1 ratio was shown to have AUC of 0.90 for CMNS versus MM, and 0.69 for CMNS versus CMS (Figure 2c, f). However, a cut-off ratio of 0.69 was shown to be 86.2% sensitive and 83% specific in differentiating CMNS from MM, and a cut-off ratio at 1.58 was 65.5% sensitive and 60.3% specific in differentiating CMNS from CMS.

## Discussion

Early detection and prognosis of CM patients with a higher risk for fatal outcome is necessary for better clinical case management and improvement of treatment outcome. It is anticipated that a better understanding of mechanisms contributing to CM-associated death will help in the development of novel adjunctive therapies to reduce mortality associated with CM. Currently there is no reliable biological test that can predict CM or its associated complications, including mortality and post recovery neurological deficits. This study reveals that in Indian CM patients ANG-2 and ANG-2/ANG-1 ratios had high levels of sensitivity and specificity (area under the ROC curve close to 1) in discriminating CMNS from MM patients. However, ANG-2 levels differentiated CMNS from CMS with high sensitivity, but moderate levels of specificity.

Other studies demonstrated ANG-1 as a good biomarker in differentiating CM from MM among Thai adults, but not in Ugandan children [24]. Yeo et al. reported that ANG-2 is a better marker of severe malaria associated deaths than lactate in Indonesian adults [15]. Among Ugandan children higher levels of ANG-1 were found to be associated with a reduced risk of death [24]. However, in our study population ANG-1 levels were not good prognostic indicators by themselves in as much as this study observed a decrease in ANG-1 levels with disease severity. Interestingly, in a recent study conducted with Malawian children, ANG-1 levels were significantly down regulated among CM patients with retinopathy compared to those without retinopathy, uncomplicated malaria patients and those with non-malarial encephalopathy [18]. It is thought that differences in the epidemiology of malaria between Asia and Africa, genetic differences in these two distinct populations and other factors (e.g. age, etc.) may explain these inconsistencies.

Consistent with earlier studies [15,18,20,21,24], this study highlight the potential involvement of ANG-1 and ANG-2 in the pathogenesis of CM in endemic setting of Central India. The breach of blood brain barrier is believed to be an important component in the pathogenesis of CM [25,26] but a recent histologic study conducted in Vietnamese adults who died of CM did not support such a hypothesis [23]. In the Vietnamese study, no significant correlation between vascular injury and death associated with CM was evident [23]. Overall, these findings suggest pathogenesis of CM is multifactorial and complex [28].

Platelets and PMP were described to enhance PRBCs sequestration to EC and this is associated with accumulation of host inflammatory cells [12,13]. Resultant inflammation may lead to CM pathology which may results in excess release of ANG-2 (from the Wiebel-Palade bodies) and loosening of endothelial tight junctions. vWF (mediate platelet/PRBCs sequestration) and IL-8 which are co-packed with ANG-2 are also seen elevated in severe malaria [12,24].

Also angiogenic expression of molecules are variable among African CM children during acute and convalescence stages [18,20,27]. Interestingly platelet and infected red blood cells which adhere to brain endothelial cells and placenta during malarial pathology have recently been reported to alter gene expression of EC and syncytiotrophoblast respectively [29,30]. This in-turn may result in EC apoptosis (via TNF, TGF-beta signaling) and inhibition of angiogenesis in placental pathology (via VEGF) [29,30]. Thus studies related to histopathology of internal vital organs in addressing oedema and heamorrhages need to be done to better understand parasite- dependent host's pathogenic/protective pathways in CM.

## Conclusions

Overall, the findings of this study suggest that plasma levels of ANG-2 and ratio of ANG-2/ANG-1 at admission are clinically informative biomarkers to predict fatal CM from MM cases albeit limited usefulness in discriminating fatal CM outcomes in a pool of CM cases in endemic settings of Central India. Combining the ANG-2 and ratio of ANG-2/ANG-1 with other suitable biomarkers (such as IP-10, sICAM-1) can help to eventually develop suitable biomarker panels for predicting CM associated fatal complications. The immunopathogenic role of ANG-2 with other important angiogenic markers in systemic vital organ dysfunction needs to be investigated in severe malaria. Further validation of these findings in patient populations from different endemic areas will help to eventually develop suitable biomarkers for diagnostic and prognostic purposes.

## Competing interests

The authors declare that they have no competing interests.

## Authors' contributions

VJ performed sample collection/processing, immunological experiments, proteomics analysis, data analysis and drafting of the manuscript. NWL and NOW participated in data analysis, drafting of the manuscript and participated in coordination of the study and technical training of field staff in India. AB, MPS participated in data analysis. ACN, PKJ participated in the design and coordination of the study, and supervised patient recruitment, clinical evaluation in India. VU, JKS and NS planned the study, participated in its design and coordination, supervision, interpretation of data and revised the manuscript for important intellectual content. All authors read and approved the final version of manuscript.
